# Mental stress due to poor organizational climate and high work commitment as predictor of 10-year registered sickness absence: a cohort study based on The Population Study of Women in Gothenburg

**DOI:** 10.1080/02813432.2025.2466177

**Published:** 2025-02-20

**Authors:** Marit Knapstad, Lauren Lissner, Cecilia Björkelund, Kristina Holmgren

**Affiliations:** ^a^ Department of Health Promotion, Norwegian Institute of Public Health, Bergen, Norway; ^b^ Department of Public Health and Community Medicine, Institute of Medicine, The Sahlgrenska Academy, University of Gothenburg, Gothenburg, Sweden; ^c^ Primary Health Care/Department of Public Health and Community Medicine/, Institute of Medicine, The Sahlgrenska Academy, University of Gothenburg, Gothenburg, Sweden; ^d^ Department of Health and Rehabilitation, Institute of Neuroscience and Physiology, The Sahlgrenska Academy, University of Gothenburg, Gothenburg, Sweden

**Keywords:** Sickness absence, psychosocial work environment, organizational climate, work commitment, cohort study, general population, register-based data

## Abstract

**Objective:**

Early identification of persons at risk for sickness absence offers an opportunity to initiate preventive measures. The aim of this study was to examine whether perceived work-related stress, measured by The Work Stress Questionnaire (WSQ), predicted registered sickness absence up to 10 years later in a general working population of women.

**Design:**

A survey-linkage study based on the 2004–2005 wave of the Swedish ‘Population Study of Women in Gothenburg (PSWG)’ and 10-year follow-up in the official MiDAS sickness absence register.

**Subjects:**

A population-based cohort of women aged 38 and 50 in 2004/2005 were invited (participation rate 59%). Participants registered as employed or self-employed were included (*n* = 396).

**Methods:**

Predictors were indicators of work-related stress (WSQ). Outcomes were number of sickness absence episodes in total (examined using negative binomial regression) and sickness absence during separate time periods of follow-up (examined in logistic regression models).

**Results:**

High perceived stress due to poor organizational climate predicted sickness absence episodes (adjusted Incidence Risk Rate (IRR_adj_): 1.99 [95% CI 1.19–3.34]). The combination of stress due to both poor organizational climate and high work commitment gave an IRR_adj_ of 2.32 [95% CI 1.26–4.26]. Examination of specific two-year time period indicated that the results were rather consistent for up to 8 years of follow-up. Additionally, low influence at work was associated sickness absence in some of the time periods examined.

**Conclusion:**

The findings indicate that perceived stress due to organizational factors at work, alone and in combination with the individual factor high work commitment, are long-lasting risk factors for sickness absence. Aligning with previous studies, the findings further suggest that WSQ might successfully aid identifying women with such elevated risk and adds that this is even true in a general population context. These findings merit further investigation in larger samples.

## Background

While work–life participation generally can promote health and is a key source for social inclusion and financial security in Western societies, it is well established that being exposed to adverse psychosocial working conditions are associated with negative health outcomes and sickness absence [1–3]. We have previously reported large increases in perceived stress in Swedish women over the past several decades [4]. Given the large-scale entry of women into the workforce during this period, it is of particular interest to understand which types of work-related stress in contemporary women are predictors of later sickness absenteeism. Furthermore, having tools that enable early identification of persons at risk for sickness absence gives the opportunity to implement preventive measures. Several psychosocial work conditions are identified as risk factors for ill health and sickness absence, such as high job demands, low job control, high effort-reward imbalance, indistinct work organization, work-life imbalance, role stress and bullying [1,2,5,6]. In particular, the widely acknowledged Demand-Control model and Effort-Reward models have shown to successfully predict negative health outcomes and sickness absence in several sectors [2,5,7–9]. However, these were mainly developed in the 1980s and 1990s in predominantly male work settings [10,11]. Thus, they may miss important stressors in the current labor market, and perhaps especially in female-dominated sectors. Knowing that the labor market in most countries, including Sweden, is highly gender-segregated, women and men as groups may both be exposed to different work stressors [1,12], and also respond differently to the same stressors [12].

The Work Stress Questionnaire (WSQ) is specifically designed to identify people at risk for sickness absence due to perceived work-related stress and aims to capture important stress factors in modern working life [13–15]. The WSQ is based on a transactional perspective of occupation [16], where the interdependence between personal characteristics and contextual factors, at work and private life, capture self-evaluated work-related stress. Changes in either one may influence the possibilities for maintaining occupational performance [17–19]. Women who have been sick-listed themselves informed the development of the questionnaire [14]. Their experiences highlighted the importance of acknowledging not only the occurrence of a work stressor, but the experience of this stressor. More specifically, they highlighted that the combination of high work commitment, such as individual demands and responsibility, and poor organizational climate, for example, indistinct leadership and conflicts at work, was decisive for future sick-listing [14]. The latter fits well with the emerging body of research finding of poor organizational climate, in terms of intolerant climate [20], conflicts at work [21,22], and injustice at work [23] to be risk factors for poor health and sickness absence. Commitment refers to a willingness to persist in a course of action [24]. To be committed to or engaged in one’s work might thus in itself be regarded a resource both from the individual’s and the employer’s perspective. Work commitment and similar measures tend to be positively related to outcomes like job satisfaction and work performance and negatively related to job turnover and related intentions [24,25]. Nonetheless, there is a downside, where highly committed workers are risking emotional exhaustion in high demanding work contexts. Mechanisms such as placing high demand on oneself, having difficulties setting limits, and a high sense of responsibility may all be contributing factors for poor health and sick leave [14,26,27]. These concepts are, however, generally examined separately, and studies are called for that include the workers’ style in examining relationships between psychosocial work factors and health, preferably with a prospective design [3,5].

Prospective studies employing the WSQ in primary care settings in Sweden [19,28], have shown that high perceived stress due to indistinct organization and conflicts at work approximately doubled the chance for sickness absence at one-year follow-up. Being exposed to such stressors and additionally experiencing stress due to individual demands and work commitment increased the risk by more than fourfold. Similar additive effects of experiencing combined stress due to both poor organizational climate and high work commitment have been observed in relation to poor self-perceived health [15], lower job well-being [29] and sickness absence [15,29]. A recently published population-based study found high stress due to indistinct organization and conflicts to be associated with incident sickness absence 12 months later [30]. Additionally, experiencing low decision latitude was found related to incident sickness absence. In summary, distinct work stressors from the WSQ are found to predict sickness absence both in primary care and general working population samples. However, like most studies on this topic, the mentioned prospective studies had relatively short follow-up time [3], limiting our knowledge about the duration of the stress-sick-leave association over prolonged time periods.

The objective of this study was to examine prospective associations between perceived work-related stress as measured by WSQ and officially registered sickness absence up to 10 years later among employed and self-employed women in the general population. More specifically, we aimed to investigate whether perceived work-related stress predicted number of registered sickness absence episodes during follow-up and occurrence of registered sickness absence during five predefined time periods during follow-up.

## Methods

The study is based on a linkage between the 2004-05 wave of the Swedish ‘Population Study of Women in Gothenburg (PSWG)’ [31] and the Swedish social insurance agency’s Micro Database for Analyzing Social insurance (MIDAS) (described below).

### The population study of women in Gothenburg (PSWG)

‘The Population Study of Women in Gothenburg’ is a longitudinal population-based study of women in Gothenburg, Sweden’s second largest city with around 500 000 inhabitants. The selection of two age groups in this study, 38- and 50-year-old women, made it possible to capture two distinct stages in women’s working lives. Cohort comparisons of 38- and 50-year-old women have been carried out periodically since 1968. This present study is primarily based on the survey wave stretching from October 2004 to April 2005. A random sample of 38-year-old and 50-year-old women, registered for census purposes in Gothenburg, was identified and invited to participate in a free health examination. In addition, 85 additional 50-year-olds were invited, who had previously been 38-year-old participants in the 1992–1993 survey wave [31]. In all, 846 women were invited, of whom seven were excluded due to difficulties in speaking and understanding Swedish, 191 declined or did not turn up and 148 could not be contacted. In total, 500 women accepted and participated in the study (60%/58% of invited 38-/50-year-olds: overall response rate 59%). Participants were more likely than non-participants to have higher income and to be born in Sweden, while there were no differences in marital status, places of living or hospital admission rates [31].

### Study sample

For the present study, we included those participants in the 2004–2005 survey described above who were identified in the MiDAS sickness absence register as employed or self-employed. We excluded those who were not employed (*n* = 67) or receiving disability pension (*n* = 17) at baseline. One participant died during follow-up and was excluded. An additional 10 women receiving disability pension during follow-up were excluded due to uncertain labor market affiliation in the transition phase between work and disability pension. Those with incomplete responses on the Work Stress Questionnaire (*n* = 9) were also excluded, leaving a total of 396 women in the present study.

### Predictor: the work stress questionnaire (WSQ)

Work-related stress at the 2004–2005 baseline was measured by The Work Stress Questionnaire (WSQ). The 2004–2005 wave was the first of the PSWG surveys to include an instrument to assess psychosocial stress specifically directed at work. The WSQ consists of 21 main questions grouped into four composite stress indicators. Two of these are perceived stress due to *poor organizational climate* and to *high work commitment*, each containing seven items. Items on organizational climate cover for instance conflict at work and unclear leadership or work tasks. Work commitment regards high degree of work engagement, high internalized work demands, difficulties in setting limits and high sense of responsibility. Respondents are first asked whether they experience this (yes, no) and those answering affirmatively are further asked to rate the degree to which they experience this as stressful on a four-point ordinal scale: ‘Not at all stressful’, ‘Less stressful’, ‘Stressful’, and ‘Very stressful’. The other two indicators are *influence at work* and *work interference with leisure time,* containing four and three items, respectively, with response alternatives on a four-point ordinal scale: ‘Yes, always’, ‘Yes, rather often’, ‘No, seldom’ and ‘No, never’. Examples of themes regarding influence at work include possibility to influence decisions and work pace, and the work-leisure interference items regards finding time for close friends and recreational activities due to work. See reference [13] for full questionnaire in English. The reliability of the WSQ has been assessed in a test–retest study with a nonparametric statistical method for evaluation of paired data, indicating a high level of reliability [13]. The items were also evaluated by a pilot sample, representing the questionnaire’s target group, who confirmed the face validity of the questions and their content [13]. The validity and reliability of WSQ has also been tested among male workers with satisfactory results [32].

Four indicators of work-related stress were operationalized based on earlier research [15,19,28]. These were (1) *influence at work*, calculated as the median of the four items for each participant and then dichotomized into low (never or seldom) and high (always or often); (2) *perceived stress due to poor organizational climate* and (3) *perceived stress due to high work commitment*, both calculated as the median of the seven items for each participant, respectively, and then dichotomized into high (confirmatory answers to stressful or very stressful) and low (not experienced, or reported not stressful or less stressful), (4) *work interference with leisure time* (calculated as the median of the three items for each participant and then dichotomized into high (always and often) and low (never or seldom).

To explore joint effects of ‘perceived stress due to organizational climate’ and ‘perceived stress due to work commitment’, we defined four possible combinations of the categories. The decision to combine the two dimensions is based both on the theoretical starting point of the transaction between personal characteristics and work-related factors [17–19] and on previous studies that support the effect of this combination [15,19,28,33]. This resulted in four categories: (1) ‘low perceived stress due to organizational climate’ and ‘low perceived stress due to work commitment’; (2) ‘high perceived stress due to organizational climate’ and ‘low perceived stress due to work commitment’; (3) ‘low perceived stress due to organizational climate’ and ‘high perceived stress due to work commitment’; and (4) ‘high perceived stress due to organizational climate’ and ‘high perceived stress due to work ­commitment’ [19,28].

### Outcome: officially registered sickness absence from MiDAS

Data from the MiDAS (Micro Data for Analyses of the Social Insurance) register was retrieved as measure of sickness absence during the period from date of participation in the PSWG 2004/05 survey baseline and ten years follow-up (2015). These data were merged with the survey data using personal identification numbers, returned anonymized. The MiDAS database is administered by the Swedish Social Insurance Agency (SSIA) and contains information on all episodes of payment of sickness benefits from SSIA. In Sweden, employers cover the first 14 days of a sickness absence episode except one qualifying day. From day 15, the sick pay is reimbursed by the SSIA. Self-employed can apply for reimbursement from day 2 of their sickness period. Thus, the MiDAS register contains data on sickness absence episodes over 14 days for employees and day 2 for self-employed. We obtained information for each participant on start and stop dates for each reimbursed sickness absence period as well as percentage sickness absence (25, 50, 75 or 100%).

Based on this information we constructed the two types of outcome variable: (1) number of registered sickness absence episodes during the full 10-year follow-up (0, 1, 2, 3, etc.) and (2) registered sickness absence at five predefined time periods during follow-up (1^st^–2^nd^, 3^th^–4^th^, 5^th^–6^th^, 7^th^–8^th^ and 9^th^–10^th^ year following baseline participation) (yes, no). These ways of operationalizing sickness absence are based on recommendations from Hensing et al. [34] and adapted according to the research question of interest and data available in MiDAS. The second outcome was included to examine the extent to which associations between the predictors and sickness absence persisted over time. Similar methods for operationalizing the sickness absence have also been employed in a Norwegian survey-linkage study on the prospective association between common mental disorders and sickness absence [35].

### Covariates

The following self-reported baseline variables were included as possible confounders, as they were previously found related to work-related stress and/or sickness absence [36,37]. These include age (38 or 50 years), educational level (primary [Swedish: Grundskola, 9 years], secondary [Swedish: Gymnasium, 12 years], higher [Swedish: Högskola/Universitet], 14 years+), occupational class (manual and lower non-manual, middle-high non-manual, miscellaneous (students, self-employed)), employer (public, private or self-employed), part time position (<80%, ≥80%), income (quintiles).

### Statistical analyses

Descriptive statistics were generated for baseline characteristics, including frequencies of perceived work-related stress and related covariates in the study sample. Associations between the indicators of work-related stress and number of sickness absence episodes were examined by means of negative binomial regression. This regression model was chosen as it can handle over-dispersed count variables, like number of sickness absence episodes. The outcomes are expressed as incidence rate ratios (IRR), which here, put simply, is an estimate of the relative difference in sickness absence episodes during follow-up among those exposed to the work stress indicator of interest compared to those unexposed. Logistic regression analysis was used to examine associations between work-related stress and having any registered sickness absence episodes during the respective follow-up periods. For all regression models, initially age-adjusted analyses were conducted before including the other covariates. We also ran sensitivity analyses, including one covariate at the time, to identify those with substantial impact on the observed associations between work-related stress and sickness absence.

There were little internal missing data, as those not completing the WSQ were excluded (*n* = 9) and the MiDAS register data were complete. Thus, only some of the covariates included in the multivariate models had missing data (work position (2.0%), income (2.8%), and occupational class (4.6%). In these analyses missing data was handled using listwise deletion.

## Results

### Baseline characteristics of the sample

The 50-year-old group slightly outnumbered the 38-year-olds (57.8% vs 42.2% of the total sample). Half of the participants were married and about two-thirds (65.4%) had children living at home. The socioeconomic position of the sample was somewhat higher than in the general working-age population [38], with around half having completed higher education and having a middle-to-high non-manual occupational position. Just above half (54.0%) worked in public sector, and 78.0% had full-time positions (Table 1).

**Table 1.** Background information about the participants (*n* = 396)*.

Variables	*n*	%
Age		
38 years of age	167	42.2
50 years of age	229	57.8
Marital status		
Married/partnership	201	50.8
Unmarried, divorced, widowed	195	49.2
Having children living at home	259	65.4
Education		
Primary school (9 years)	22	5.6
Secondary school (12 years)	152	38.4
Higher education (14+ years)	222	56.0
Occupational class		
Manual and lower non-manual	164	41.4
Middle-high non-manual	189	47.7
Misc. (students, self-employed)	25	6.3
Missing	18	4.6
Employer		
Public	214	54.0
Private or self-employed	182	46.0
Work position		
Full time (≥80%)	309	78.0
Part time (<80%)	79	20.0
Missing	8	2.0
Income (in 1000 SEK, mean (SD))	267.2	117.3
Number of registered sickness absence episodes during 10 years follow-up		
0	194	49.0
1	90	22.7
2	46	11.6
3	22	5.6
4+	44	11.1
Registered sickness absence episode in specific time periods (≥ 1 episode à > 14 days)		
1^st^–2^nd^ year	94	23.7
3^rd^–4^th^ year	71	17.9
5^th^–6^th^ year	73	18.4
7^th^–8^th^ year	81	20.5
9^th^–10^th^ year	78	19.7

*Study sample included participants in the 2004–2005 wave of The Population Study of Women in Gothenburg, Sweden, listed in the MiDAS register as employed or self-employed at baseline.

### Baseline frequencies of work-related stress

The most common work stress indicator among the participants was high level of work-leisure interference, reported by one-third. One in four reported high stress due to work commitment, one in five low influence at work and around one in ten high stress due to poor organizational climate (see Table 2 for more details). When combining responses regarding organizational climate and work commitment, the majority (72.7% [95% CI 68.1–76.9]) were categorized as experiencing low stress on both these aspects, whereas 6.3% [95% CI 4.3–9.2] were categorized as experiencing high stress in regarding both.

**Table 2.** Baseline frequency (%) of perceived work stress in the study sample of working women in the population study of women in Gothenburg, Sweden, 2004–2005 (*n* = 396).

	*n*	%	95% CI
Low influence at work	81	20.5	16.8–24.7
High stress due to poor organizational climate (OrgClim)	35	8.8	6.4–12.1
High stress due to work commitment (WorkCom)	98	24.7	20.7–29.3
High level of work-leisure interference	131	33.1	28.6–37.9
Combinations of perceived stress due to poor organizational climate (OrgClim) and high work commitment (WorkCom)			
Low stress OrgClim and low stress WorkCom	288	72.7	68.1–76.9
High stress OrgClim and low stress WorkCom	10	2.5	1.4–4.6
Low stress OrgClim and high stress WorkCom	73	18.4	14.9–22.6
High stress OrgClim and high stress WorkCom	25	6.3	4.3–9.2

### Associations between perceived work-related stress and number of sickness absence episodes during the total follow-up period

In total, 51.0% of the participants had at least one episode of registered sickness absence (i.e. episodes of > 14 days) during the 10-year follow-up period. Median number of registered sickness absence episodes during follow-up was 1 [IQR 0–2]. Table 3 details the incident rate ratio (IRR) for number of registered sickness absence episodes during the 10-year follow-up by each of the work stress indicators.

**Table 3.** Number of sickness absence episodes during 10-year follow-up by perceived work-related stress among working women in the Population Study of Women in Gothenburg, Sweden, 2004–2005. Negative binomial regression.

	Model 1^a^		Model 2^b^	
	IRR	95% CI	IRR	95% CI
Low influence at work	1.22	0.85–1.77	1.10	0.74–1.64
Perceived stress due to poor organizational climate	**1.96**	**1.20–3.20**	**1.99**	**1.19–3.34**
Perceive stress due to work commitment	1.20	0.85–1.69	1.16	0.80–1.68
High work-leisure interference	0.92	0.66–1.27	0.97	0.68–1.34
Combinations of perceived stress due to organizational climate (OrgClim) and perceived stress due to work commitment (WorkCom)**				
Low stress OrgClim and low stress WorkCom	1.00		1.00	
High stress OrgClim and low stress WorkCom	0.95	0.36–2.48	1.10	0.41–2.93
Low stress OrgClim and high stress WorkCom	0.83	0.56–1.24	0.81	0.53–1.24
High stress OrgClim and high stress WorkCom	**2.29**	**1.30–4.03**	**2.32**	**1.26–4.26**

^a^Adjusted for age.

^b^Adjusted for model 1 + education, occupational class, employer, income and employer (public or private/self-employed).

Statistically significant results at alpha >.05 in bold.

High perceived stress due to poor organizational climate gave 1.96 (95%CI 1.20–3.20) times higher incident rate for sickness absence episodes compared to low stress (age-adjusted). The predicted mean number of sickness absence episodes was 2.31 (95%CI 1.24–3.38) for the high stress and 1.18 [95%CI 1.00–1.36] for the low stress group (not shown in table). The fully adjusted model hardly changed the estimates.

The combination of high perceived stress due to both poor organizational climate high work commitment gave a 2.29 (95%CI 1.30–4.03) times higher incident rate for sickness absence episodes, compared to the low stress group (age-adjusted). The predicted mean number of sickness absence episodes was 2.79 (95%CI 1.30–4.29) for the former and 1.22 (95%CI 1.00–1.43) for the latter group. In the fully adjusted model, the IRR for sickness absence episodes remained largely stable. Separate adjustments showed that inclusion of income as a covariate yielded a marginally increased point-estimate for the association between stress and absence episodes.

The remaining work stress indicators were not statistically significantly associated with number of sickness absence episodes (Table 3 for details).

### Associations between perceived work-related stress and number of sickness absence days during separate time periods 0–10 years following baseline

Across each two-year period of follow-up the proportion having sickness absence and median length of absences were rather consistent; Around one in five (18.4-23.7%) was registered least one sickness absence episode per period (Table 1). Among those with sickness absence, the median (IQR) number of gross sickness absence days were 44.5 (128), 45.0 (83), 40.0 (67), 53.0 (113) and 80.0 (167), respectively, during the 1^st^–2^nd^, 3^rd^–4^th^, 5^th^–6^th^, 7^th^–8^th^, 9^th^–10^th^ year follow-up year.

Perceived low influence at work, high perceived stress due to poor organizational climate, and combined high perceived stress due to poor organizational climate and high work commitment were all associated with sickness absence in both shorter (1^st^–2^nd^ year) and longer term (5^th^–6^th^ and 7^th^–8^th^ year) follow-up (Table 4 for details). The strength of these associations was rather stable across period studied, with odds ratios around 2 for low influence at work, 2.5 for poor organizational climate and 2.5–3.5 for the combined stress situation, compared to the respective low stress reference groups (Table 4).

**Table 4.** Associations between perceived work stress and subsequent registered sickness absence among working women in the population study of women in Gothenburg, Sweden, 2004–2005.

	1^st^-2^nd^ year*		3^rd^-4^th^ year*		5^th^-6^th*^		7^th^-8^th*^		9^th^-10^th*^	
	OR	95%CI	OR	95%CI	OR	95%CI	OR	95%CI	OR	95%CI
Low influence at work										
Model 1^a^	**1.98**	**1.16–3.37**	1.41	0.77–2.57	**1.92**	**1.08–3.04**	**2.07**	**1.19–3.60**	0.90	0.48–1.68
Model 2^b^	**1.83**	**1.03–3.24**	1.22	0.64–2.33	1.78	0.96–3.32	**2.00**	**1.11–3.61**	0.71	0.35–1.42
High stress owing to organizational climate										
Model 1^a^	**2.34**	**1.13–4.81**	1.66	0.74–3.73	1.89	0.86–4.13	**2.55**	**1.22–5.32**	1.46	0.65–3.25
Model 2^b^	2.14	0.98–4.69	1.79	0.77–4.14	**2.32**	**1.01–5.36**	**2.87**	**1.32–6.24**	1.39	0.58–3.28
High stress owing to work commitment										
Model 1^a^	1.50	0.90–2.51	0.95	0.52–1.72	1.40	0.80–2.46	1.37	0.80–2.36	0.74	0.40–1.35
Model 2^b^	1.54	0.89–2.70	0.93	0.49–1.76	1.49	0.81–2.76	1.50	0.84–2.67	0.76	0.39–1.47
High work-leisure interference										
Model 1^a^	1.00	0.61–1.65	0.58	0.33–1.06	0.78	0.45–1.37	0.94	0.56–1.59	0.76	0.44–1.31
Model 2^b^	0.88	0.51–1.50	0.58	0.31–1.11	0.85	0.46–1.58	1.13	0.65–1.98	0.88	0.49–1.57
Combinations OrgClim and WorkCom:										
L. stress OrgClim & L. stress WorkCom	1.00		1.00		1.00		1.00		1.00	
H. stress OrgClim and L. stress WorkCom										
Model 1^a^	2.29	0.62–8.41	0.47	0.06–3.83	0.51	0.63–4.13	1.88	0.47–7.55	2.47	0.67–9.10
Model 2^b^	2.18	0.57–8.27	0.49	0.06–4.03	0.59	0.07–4.99	1.92	0.46–7.99	2.53	0.67–9.61
L. stress OrgClim and H. stress WorkCom										
Model 1^a^	1.29	0.71–2.34	0.62	0.29–1.32	1.02	0.52–2.00	1.03	0.54–1.98	0.69	0.67–1.40
Model 2^b^	1.40	0.74–2.62	0.57	0.25–1.29	1.00	0.47–2.11	1.08	0.54–2.18	0.76	0.36–1.62
H. stress OrgClim and H. stress WorkCom										
Model 1^a^	**2.54**	**1.08–5.96**	2.11	0.86–5.14	**2.70**	**1.13–6.46**	**2.88**	**1.23–6.77**	1.01	0.36–2.80
Model 2^b^	2.37	0.92–6.11	2.30	0.89–5.94	**3.48**	**1.33–9.08**	**3.48**	**1.39–8.73**	0.89	0.28–2.79

Five separate time periods.

^a^ Adjusted for age.

^b^ Adjusted for model 1 + education, occupational class, income and employer (public/private).

Statistically significant results at *p* <.05 in bold.

Adjusting for confounders attenuated some of the observed associations. More specifically, in the low influence at work model, the estimates were no longer statistically significant after full adjustment for year 5–6. Separate adjustments showed that controlling for income or employer (public/private) attenuated the estimate. In both the organizational climate model and the combined stress model, the fully adjusted model gave nonsignificant results for year 1–2. Again, separate adjustments showed that income was the single variable attenuating the confidence interval towards a statistically non-significant association. Notably, the estimates on the contrary increased after adjustments in these models in year 5–6 and 7–8.

No statistically significant associations with sickness absence were observed for perceived stress due to work commitment and work-leisure interference. And no statistically significant associations were found in the 3^rd^–4^th^ and 9^th^–10^th^ year follow-up.

## Discussion

### Main findings

In the current study, we aimed to examine whether various indicators of perceived work-related stress, as measured by the Work Stress Questionnaire (WSQ), could predict future registered sickness absence in a general working population of women, in shorter, longer, and very long-term (up to 10 years following baseline). The data showed that women reporting high stress due to poor organizational climate, like indistinct organization and conflicts at work, had both more sickness absence episodes in total and increased odds for having sickness absence at both shorter (1^st^–2^nd^ year) and longer term (5^th^–8^th^ year) follow-up. The combination of stress due to both poor organizational climate and high individual demands and commitment further increased this risk. Reporting low influence at work also predicted sickness absence in some of the timespans, but was not associated with number of sickness absence episodes during the total follow-up.

### Interpretation of findings

#### High perceived stress due to poor organizational climate, alone and in combination with high work commitment, predicted sickness absence

The finding that perceived stress due to indistinct organization and conflict predicted sickness absence, especially pronounced in combination with high work commitment, is consistent with previous studies using the WSQ in different study designs and settings [15,19,28]. Perceived poor organizational climate, like low role clarity and organizational injustice, is similarly found to be a determinant of subsequent sickness absence and other negative health outcomes in other studies [5,39–42]. Observed association between low influence at work and sickness absence fits well with theoretical and empirical studies related to the Job Demand-Control model and negative health outcomes [2,5,7,8]. The current study adds new knowledge by showing that these aspects, as measured by the WSQ, also predict sickness absence beyond one-year follow-up in a general working population sample. Our findings might illustrate that people can experience problems with work-related stress long before even contemplating sick leave. Some ignore their symptoms and carry on with their lives for a long time before they consult health care. When they eventually do, it seems most important to identify work-related stress to prevent the risk for sick leave and to promote a sustainable working life [14,19,28,43]. Possibly reflecting this gradual process, stronger indications of work-related stress was found in a sample of primary care patients having sought care for health complaints [19], than in the current, general population-based sample. For instance, the prevalence of stress due to poor organizational climate was double as high in the primary care sample [19] compared to the current, population-based sample, where one in five reported this problem in the former and one in ten in the latter. Similarly, one in four in the current sample and half of the women in the primary care reported stress due to high work commitment.

We hypothesized that perceived stress due to both poor organizational climate and high work commitment would yield a stronger risk of sickness absence than either of these indicators *per se* [44]. This seemed in general to be supported by the data, as the combined stress group had somewhat elevated risk of sickness absence across all tests performed. It should, however, be noted that the confidence intervals were overlapping, making the evidence of an additive effect somewhat uncertain. Most of the participants reporting stress due to poor organizational climate also reported high work commitment (25 of 35 participants). Similar findings pointing towards such additive effects are nonetheless observed in all previous studies using the WSQ [19,28,29], as well as among both women and men in a population-based study examining similar work stress situations [29]. Together, the findings may indicate that being highly committed to one’s work, in fact could make one extra vulnerable in situations where the work climate is characterized by conflict or indistinct leadership [30]. Whether such effects are ‘merely’ additive or also multiplicative (i.e. effect moderation) would be interesting to examine in future studies with higher statistical power.

#### Interpretations of null findings

Aligning with some [15,28], but not all [19], previous WSQ studies were the non-significant associations between perceived stress due to individual demands and commitment and high degree of work-leisure interference, and later sickness absence. However, a systematic review reported moderate evidence that high perceived work-family conflict predicts subsequent sickness absence [45]. Both work commitment [25] and high work-leisure interference [46] might be more common among women in higher socioeconomic positions. As a group, these may have both other life conditions protecting against ill health, and more flexible jobs making it possible to adjust work tasks to work capacity. These factors could both reduce the need or risk of sickness absence [47,48]. Interestingly, though, we did not find direct support of such interpretation in our data, as neither the work commitment nor the work-leisure interference indicators were associated with the included socioeconomic indicators (occupational class, income and education). Regarding people with high work commitment, it might be that they manage to cope if the organizational climate is at a favorable level. Another interpretation is that WSQ-questions are more suitable to detect those with problematic work-leisure interference in primary care contexts [19], where the instrument was developed and where the person already has sought help due to emerging symptoms. Furthermore, the range of sickness absence occurrences were low in the sample, as expected for a population sample, and may have influenced the ability to detect effects. This may also contribute to explaining why stronger associations have been found in patient samples, where incidence rates are likely to be higher and thus yield more variability to capture effects [19].

#### Work-related stress predicted sickness absence in some, but not all follow-up periods

Work-related stress predicted sickness absence in both shorter and longer term, even up to 7 to 8 years after reported exposure. Sickness absence was notably not predicted in all (3^rd^–4^th^, 9^th^–10^th^ year) of the follow-up periods, which may be explained by a combination of factors. For instance, during the follow-up period, changes in the Swedish sickness insurance scheme have been made. In 2008, an upper limit of 365 sick leave days was fixed. In addition, the so-called rehabilitation chain was introduced, which is a well-defined process with different steps to be taken at certain points in time to promote people returning to work [49,50]. These changes were mainly directed at reducing long-term sick leave. These new rules might have influenced sick leave patterns during the 3^rd^ and 4^th^ years after baseline.

It is nevertheless conceivable that distinct processes are driving the observed associations in shorter versus longer term [51,52]. For instance, the observed work stress at baseline might be more directly related to, and possibly a cause of, the sickness absence in the shorter term (1^st^–2^nd^ year), while other (unmeasured) intervening factors are likely to contribute to drive an association with sickness absence in the longer term. We have no information on the persistency of work-stress exposures and both within-organization change and change of workplace are conceivable over such long follow-up time. Interestingly, a recent study found differences in associations between organizational injustice and sickness absence by whether or not the employee felt “locked in” at the workplace [53]. It would thus be interesting and informative to have measured both work-related stress and sickness absence at several time points to examine in more detailed how for instance prolonged expose to work stress affect sickness absence and whether the factors influence each other reciprocally [51]. Few such studies exist, and to our knowledge none have examined work stress and sickness absence with a ten-year follow-up. As such, though the mechanism is unknown, it is still highly noteworthy that perceived work-related stress may be a red flag of poor health outcomes up to eight years later.

## Strengths and limitations

The main strengths of the study were the population-based prospective design with very long-term follow-up period in high-quality registries. Sweden’s registry data are highly complete and accurate, yielding practically no attrition and hence precluded common problems of selective attrition [51]. Objective data additionally prevents problems of recall or report bias potentially related to self-report of sickness absence [54,55]. Both sources of bias are particularly relevant considering the length of the follow-up. The long follow-up also enabled us to explore relevant time lags between exposure to work-related stress and health outcomes [51]. Other strengths include the use of a work stress measure specifically designed for the purpose of interest in the study, and which is tested for reliability and face validity, and finally the use of a general working-age population sample, which increase generalizability of findings. It is furthermore an important strength that sickness absence was operationalized in two different ways to capture both the overall and period-specific effects.

Some limitations should be noted. The sample was rather small, constraining statistical power. This precluded more fine-tuned examinations of relations between work stress and aspects of sickness absence (such as time to first absence and length of absences). The risk of type two errors also increases, and precision of estimates lower as indicated by wide confidence intervals. For instance, we did not find an expected association between education level and WSQ and sickness absence. This might be explained by having few cases having the lowest education level (*n* = 22). Furthermore, as 20% (*n* = 78) had a part-time position and a similarly large group had very low income, one might question the degree of exposure these have experienced, which may have impacted on the observed associations. Furthermore, we do not have information about how other factors during follow-up may have affected the relationship between work stress and sickness absence, reducing our possibilities of making causal inference [51]. Related, though we adjusted for important potential confounders, residual confounding cannot be precluded. For example, psychosocial conditions outside work (such as social support) and health-related behavior (such as alcohol consumption) may act as both confounders, moderators and mediators of the complex work stress-sick leave association. In sum, the observed associations should be interpreted with caution and preferably be replicated using a larger sample and a more comprehensive model. As in most other survey studies, non-participation is a challenge. In the current study participants had higher socio-economic positions and were more likely to be born in Sweden than the non-participants, while there were no difference by participation in marital status, places of living or hospital admission rates [31]. As associations are less prone to selective participation bias than prevalence estimates, less emphasis should be placed on the prevalence estimates presented.

## Conclusions

To enable effective preventive measures for sickness absence, it is an important, albeit complicated, task to identify people at risk. The findings of the current study do, in concert with previous studies of the WSQ, indicate that the WSQ instrument might serve useful to identify women with elevated risk of sickness absence in the general working population. The results underscore the health risk of experiencing poor organizational climate. Moreover, being highly committed to one’s work and having high individual demands might make one extra vulnerable when the work climate is characterized by conflict or indistinct leadership. This is of special relevance in view of secular increases in perceived stress evidenced in contemporary female populations.

## Data Availability

The datasets generated and/or analyzed during the current study are not publicly available due to ethical restrictions and personal data protection but are available from the corresponding author on reasonable request.
